# Adolescent Perspectives on the Pharmacy-Based T-EVER (Teen E-Cigarette and Vaping Educational Resource) and Its Potential Impact on Youth Vaping

**DOI:** 10.3390/pharmacy12040101

**Published:** 2024-06-28

**Authors:** Grace C. Klubertanz, McKennah J. Matulle, Jenny S. Li, Olufunmilola Abraham

**Affiliations:** Social and Administrative Sciences Division, School of Pharmacy, University of Wisconsin-Madison, Madison, WI 53705, USA; gklubertanz@wisc.edu (G.C.K.); mmatulle@wisc.edu (M.J.M.); jenny.li@wisc.edu (J.S.L.)

**Keywords:** e-cigarette, vaping, adolescents, educational tool, pharmacist intervention

## Abstract

Background: While public health efforts have made tobacco smoking near obsolete among adolescents, vaping products are quickly taking their place. With the negative health consequences looming ahead of young vapers, there is a desperate need to curb youth vaping. Adolescents want to be actively engaged in their health which creates space to educate on vaping in this population segment. Methods: From January to May 2023, 35 adolescents aged 11–18 participated in interviews to assess the investigator-developed Teen E-cigarette and Vaping Educational Resource (T-EVER). All the interviews were recorded and transcribed for independent analysis by two study team members. Results: The participants liked the T-EVER, indicating they were engaged in the content. However, some participants wanted more information. The participants wanted health professionals to educate them on vaping but were worried about the potential barriers facing the implementation in community pharmacy settings. Conclusions: Adolescents want to learn about vaping, but there are limited opportunities to do so. The T-EVER is designed to educate youth about vaping. This tool was well received and has the potential to be used by pharmacists as a vaping prevention and cessation intervention. More research is required to discern the true scope of the pharmacist’s role in using an educational tool to address adolescent vaping.

## 1. Introduction

### 1.1. Prevalence of Adolescent Vaping

With the introduction of vape products in the United States, there was a drastic shift in nicotine use among young people. While smoking rates have plummeted among adolescents, studies have shown a 900% increase in youth vaping rates during this time from 3% in 2011 to 27% in 2015 [1]. This comes with minimal surprise to most since the marketing of electronic cigarettes (e-cigarettes) has increased the prevalence of vaping among youth by targeting them as a primary audience. One study indicated that during the rise of vape products, 78% of middle school and high school students were exposed to at least one advertisement promoting vaping [2]. In addition, e-cigarette manufacturers have intentionally incorporated flavorings into vape products to make them more appealing to adolescents, encouraging their use among this vulnerable population segment [3].

More recently, we have continued to see an increase in adolescent vaping rates, leading to a peak of 3.6 million high schoolers who admitted to vaping in 2018, reaching epidemic rates of youth vaping prevalence [4]. As high school vaping rates have increased, trends have identified that adolescents are initiating e-cigarette use earlier, with growing rates of middle school vaping as well [5]. The increasing rates and earlier initiation of youth vaping have led researchers to explore different regulations and interventions aimed at addressing the adolescent vaping epidemic [6]. Youth vaping rates are increasing more rapidly among Canadian and American adolescents compared to adolescents in England. This may be attributed to the nicotine limit enforced by English law, indicating that monitoring e-cigarette use and increasing regulations may be needed to address adolescent vaping rates in the US [7]. However, researchers are concerned that adolescents may be further enticed to use these products due to authorities deeming them off-limits [6].

### 1.2. Vaping Consequences

While e-cigarettes are widely accepted as a safer alternative to smoking tobacco, there are alarming long-term health consequences that cannot be ignored [8]. Nicotine is an addictive substance that is found in high concentrations in e-cigarettes [1]. This is especially concerning for individuals who are initially naive to nicotine, like adolescents who initiate use of only e-cigarettes [9,10]. People who vape and do not use e-cigarettes as a tobacco cessation aid are subjecting themselves to the addictive nature of nicotine [1]. In addition to nicotine, the chemicals found in the aerosol inhaled from e-cigarettes pose great harm to many organ systems in the body [10].

Lungs are vulnerable to these aerosolized chemicals, as seen by e-cigarette users developing e-cigarette, or vaping, product use-associated lung injury (EVALI). The symptoms of EVALI have been linked with 2807 hospitalizations [11]. This lung injury is correlated with many toxins from e-cigarette aerosols, such as propylene glycol and carcinogenic compounds like formaldehyde, which alter lung function [10,12]. Additionally, e-cigarettes function by heating up metal coils in the device, exposing users to metals like tin and aluminum when they inhale. One review found a correlation between vaping and the development of asthma and chronic obstructive pulmonary disease in youth, introducing potential long-term lung damage due to the manifestation of these disease states [13].

In addition to physical health concerns, nicotine addiction from vaping has often led to the gateway use of traditional combustible cigarettes and other substances [14,15]. Research has shown that adolescent e-cigarette users are more likely to experimentally or consistently smoke cigarettes later in life compared to non-e-cigarette users [16]. As a mental health consequence, adolescents who vape have exhibited cognitive developmental effects that negatively impact their memory and attention [14].

### 1.3. Current Trends in Adolescent Vaping Prevention

**Figure A1:**
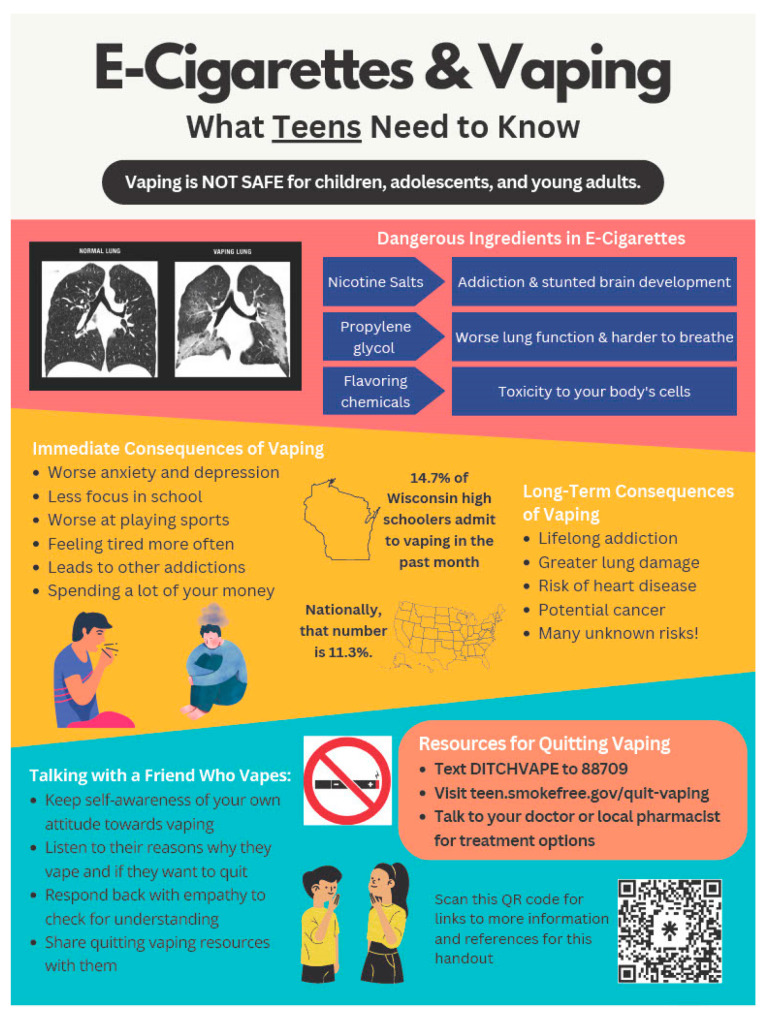
Teen E-cigarette and Vaping Educational Resource (T-EVER).

Current trends in primary practice indicate that physicians do not typically screen adolescents for e-cigarette use and vaping during visits, potentially due to a lack of physician awareness of the vaping epidemic among youth [17,18]. To help bridge the gap of vaping interventions for youth, one study found that enlisting the help of a pharmacist can increase the likelihood of helping e-cigarette users quit vaping [19]. This shows the potential role that pharmacists can play in screening and assisting adolescents with vaping cessation. To support the use of an educational handout, research has shown that familial communication and educational public health campaigns could be successful avenues to engage youth in learning about the dangers of vaping [20,21]. To address this gap in adolescent e-cigarette education, the Teen E-cigarette and Vaping Educational Resource (T-EVER) (Appendix A: Figure A1) was created to allow an avenue for pharmacists to teach adolescents about the dangers of e-cigarette use. This study aims to describe adolescent perspectives on the T-EVER, its potential impact on youth vaping, and the role of pharmacists in addressing adolescent e-cigarette use.

## 2. Materials and Methods

### 2.1. Instrument Development

The T-EVER is an adolescent-tailored infographic on adolescent vaping developed with feedback from the pharmacists, adolescents, and parents from previous studies that have yet to be published [22]. The infographic referenced vaping resources from the Centers for Disease Control, National Council for Mental Wellbeing, Truth Initiative, and Smokefree.gov Initiative [22,23,24,25,26]. The vaping educational tool provided an image of a vaping lung vs. a healthy lung, dangerous ingredients in e-cigarettes, immediate and long-term consequences of adolescent vaping, Wisconsin statistics on adolescent e-cigarette use, talking with a friend who vapes, and resources for quitting vaping. The references along with additional resources were also linked in a QR code at the bottom of the educational handout.

### 2.2. Sample and Recruitment

Participants were recruited through various methods from January to May 2023. At twelve Wisconsin community pharmacies, recruitment flyers were distributed by the pharmacy staff at the pharmacy. Two of these twelve pharmacies sent recruitment emails via pharmacy email newsletters, with one pharmacy having sent the recruitment email out four times, while the other pharmacy sent the email out two times. A study team member distributed recruitment flyers to patients at three of these twelve pharmacies for 1–2 weeks each.

The recruitment flyers shared in person possessed a QR code that linked to a Qualtrics survey allowing the potential participants to fill out their name and email if interested in participating in the study. A study team member would then email the potential participants with a link to another Qualtrics survey that contained the screening, consent, and assent forms for the parents and adolescents to complete. The virtual recruitment email newsletters provided a direct link to the Qualtrics survey containing screening, consent, and assent forms. The eligible participants were adolescents ages 12 to 18 years participating with one of their parents. All the participants must have exhibited English fluency, access to the internet, and access to a device that could use Zoom Video Communications. The participants who fully completed the screening, consent, and assent forms were then emailed up to three times by a study team member to schedule a Zoom meeting for the study session.

### 2.3. Data Collection

All the study sessions were conducted via Zoom at the time and location of the participant’s choosing. A study team member conducted all the virtual study sessions with both the adolescent and parent participants independently from one another. Each participant had their own session with a study team member to complete the pre- and post-surveys, along with the semi-structured interview.

To gather feedback on the T-EVER, the interviewer sent the participants a link to a Google Drive PDF version of the T-EVER so that the participants could review it on their own devices for as long as they needed. Adolescent participants were interviewed on the T-EVER content, the prospective modes of dissemination by community pharmacies, and the role of pharmacists in addressing adolescent vaping. The interviews were audio-recorded and transcribed verbatim for data analysis.

Upon the completion of the study session by both the adolescent and the parent, each pair was sent a $30 Amazon e-gift card. In total, the study team collected pre- and post-survey responses from 35 adolescents and 35 parents in Wisconsin. This paper focuses solely on the findings from the adolescent interviews.

### 2.4. Data Analysis

All the adolescent interviews were qualitatively analyzed independently by two study team members not involved in the interview process. An inductive approach was applied by all the analysts for content and thematic analysis. This allowed the most prevalent themes and ideas to emerge from the data collected. The study team members analyzed the participant responses using the NVivo 13 software and compared the findings using Excel Version 16.80 to find the most prevalent ideas presented.

## 3. Results

### 3.1. Demographics

Although the age criterium was 12 to 18 years, adolescents in this study ranged from 11 to 18 years with a mean age of 14.4 years (SD 1.87). The most common race or ethnicity of the participants was White/Caucasian (74.3%). The non-White participants primarily identified as multiple ethnicities. The complete demographic data can be found in Table 1.

### 3.2. Main Themes

A total of 35 adolescents shared their opinions on the usefulness and implementation of the T-EVER in e-cigarette and vaping interventions in the community pharmacy setting. Through this study, five main themes emerged: (1) adolescent knowledge and perceptions of vaping, (2) adolescent preferences of the T-EVER, (3) T-EVER feedback, (4) barriers to pharmacy-led T-EVER implementation, and (5) pharmacy role in e-cigarette education. Additional quotes are listed verbatim by subtheme in Appendix B, Table A1, Table A2, Table A3, Table A4 and Table A5.

### 3.3. Theme 1: Adolescent Knowledge and Perceptions of Vaping

#### 3.3.1. Adolescent Prior Vaping Knowledge

When asked about what they knew about vaping prior to assessing the T-EVER, most participants shared about the vaping health concerns they had previously learned. The overwhelming sentiment shared by most participants was that vaping was addictive. In addition, many participants listed a wide variety of the negative health consequences of vaping. The interviews highlighted the negative consequences posed to the lungs and other organs like the brain, as well as the potential increased risk of cancer. Most participants shared that they learned about vaping and its health impacts from their school curriculum. However, there were varying opinions on the success and completeness of this curriculum, and many participants acknowledged they still wanted to learn more.

“I know it’s not good for you because it has addictive chemicals and bad, like, metals and flavors in it.”—Adolescent 25

“I heard about this thing called popcorn lung that you can get from, from it. I just—I don’t know any specific stuff that you get from vaping, but I just know, it’s, like, I don’t know, bad for you, I guess.”—Adolescent 10

“[We] talked about it a little in health, and like, that it’s like, not good for like, teens.”—Adolescent 13

#### 3.3.2. Perceived Prevalence of Adolescent Vaping

Over half of the adolescent participants perceived that vaping was common among their peer counterparts, but very few acknowledged that they personally vaped. The participants shared varying experiences of witnessing peers vape. For example, some indicated that they have heard that their peers vape but do not see it in person; whereas others shared that they have witnessed peers vaping, most often seen at school.

“I’m going to have to go very common, because I have at least 15 kids that I’m acquainted with, but if I say, like, I have those 15 kids I’m acquainted with that vape. But then to add on, I know of a bunch of other people that do vape. I’m not acquainted with them, but I do know of them vaping.”—Adolescent 19

“I don’t see a lot of people using it in school, but I’ve definitely heard that a lot of the certain kids do use it outside of school.”—Adolescent 10

“I’ll walk into a school bathroom, whole bunch of people vaping, you know, it’s very, very common, and it shouldn’t be, you know, it’s, yeah, it’s bad.”—Adolescent 6

#### 3.3.3. Vaping Culture among Adolescents

Surprisingly, almost all the participants indicated that they were either against other youth vaping or knew it was unsafe for their peers to vape. A few participants proceeded to share some of the reasons why they think their peers vape, but then admitted their peers were misguided on the risks and benefits of vaping. Even among these misconceptions about the false benefits of vaping, the participants agreed that underage e-cigarette use was highly addictive and dangerous, creating lasting consequences for adolescents into adulthood.

“I really don’t think, kids getting, the younger and younger kids getting into it, like their bodies won’t recover as well, I feel, and like it could really mess them up, and I don’t think it’s good for teenagers to vape, especially like, if they’re freshmen and just coming into high school, like still 14 years old, you know.”—Adolescent 8

“They think it’s helping with whatever they’re going through, but it’s not.”—Adolescent 3

### 3.4. Theme 2: Adolescent Preferences of the T-EVER

#### 3.4.1. Desired Platform for the T-EVER

Most participants perceived that the current platform for the T-EVER, a physical handout, was an inadequate mode to reach the desired audience. Instead, there was a desire for the handout to be available in a digital format because it would increase accessibility to the T-EVER tool. Most notably, the adolescent participants wanted to see this educational handout on social media platforms commonly used by adolescent audiences. A less commonly mentioned suggestion included transforming the T-EVER into a video that would allow for increased ease of viewing.

“I think that maybe, putting it on, putting it on popular websites might help, or definitely, like, putting it on social media, that kind of stuff, could be important, and just kind of having it in a lot of places really is the best way, then, because then you’re guaranteed to see it, and if you don’t know much about vaping, it’s going to help you understand it more.”—Adolescent 28

“A video probably, like, like, a short, like, animated video that, like, tells the effects of vaping.”—Adolescent 34

#### 3.4.2. Adolescent Use of the T-EVER

Most participants said that they would share the T-EVER and the information they learned from it with their families. Specifically, the participants found it helpful to discuss the immediate consequences of vaping, as they are more tangible than the long-term consequences. A few participants shared that the information presented in the T-EVER was helpful for adolescents, as it guided the discussion of vaping for their families, indicating that many adolescents want to engage their families in conversations about the safety and prevalence of vaping.

On the other hand, when the participants were asked how they wanted to review the T-EVER, most preferred to read the handout on their own rather than have a pharmacist go through the T-EVER with them. However, some participants still wanted a pharmacist to take an active role in their review of the T-EVER. Some participants shared that they wanted a pharmacist to lead them in the discussion, while others preferred for the pharmacist to be available to help them if they had questions while reviewing the T-EVER.

“It has a lot of, like, simple facts on it stated clearly, so that would make it easier to talk about it.”—Adolescent 3

“[I] would rather just review it on my own, but I think it would be more effective if the pharmacist read over it.”—Adolescent 32

“I would probably rather have, like, someone go through it with me, because if I just get it on my own, I’m way less likely to actually read through it and, like, process it all.”—Adolescent 9

#### 3.4.3. T-EVER Availability in the Community

The participants shared that while the T-EVER was a great tool to facilitate learning about youth vaping, the strategic dissemination of the tool is necessary to reach the target audience. Most participants in this study indicated that the T-EVER needed to be distributed in the locations of a high density of adolescent activity. While a lot of ideas were given by the participants, the most common suggestions were at their schools, health care facilities, and community locations.

“I think it would be good at, like, maybe, like, pharmacies, schools. I definitely think it’d be good at definitely schools, or even just like, just, just places, like, I don’t know, maybe, like, a doctor’s office or something like that, or even just, like, in public, public places too, just for it to be available.”—Adolescent 10

### 3.5. T-EVER Feedback

#### 3.5.1. Positive Feedback

The participants in this study more commonly shared positive feedback regarding the T-EVER compared to negative feedback. Specifically, the participants liked that the colors and pictures were engaging. They also shared that the facts and percentages were thought-provoking and attention-grabbing. Additionally, most participants shared that the information was presented in a way that was easy to understand. This feedback indicates that the T-EVER has a dynamic design that is valuable and easy to understand, ultimately proving to be beneficial to the adolescent community.

“I like the colors. That’s probably, like, a little strange, but I like the colors and the design of it, and I think it’s, it’s very appealing to me.”—Adolescent 33

“I liked that it had, like, statistics and, like, numbers and stuff and, like, of Wisconsin, and then the whole US.”—Adolescent 4

“Um, I like—it was, like, um, easy to read, like, the information, like, it kind of had a lot on it and it was easy to understand, and, like, I don’t know, like, it was, like, like, a lot in like, a little bit.”—Adolescent 14

#### 3.5.2. Negative Feedback

There were some negative comments on the T-EVER that could help improve this handout for future use. The primary concern was that the participants wanted more information on e-cigarettes and vaping. Specifically, the participants wanted more information on the long-term consequences of vaping and the chemical contents of e-cigarettes. This demonstrates that adolescents are interested in learning more about the reasons why they should not be vaping. Furthermore, some participants shared that the reading level for the T-EVER was too advanced, indicating that future versions of the T-EVER could be modified to a more appropriate reading level for all the target audiences.

“I would have liked to have more specific information. I know it kind of gets crowded then with a lot of words, but that would be my input.”—Adolescent 31

“I thought it was a good handout. Um, I mean, some of the questions I was wondering about because, well, I—some of them I don’t understand the words or something. I’m younger and stuff like that. But most of it I did understand, and I thought it was useful. Yeah.”—Adolescent 15

### 3.6. Theme 4: Barriers to Pharmacy-Led T-EVER Implementation

#### 3.6.1. Barriers to Disseminate the T-EVER to Adolescents

The participants were asked to evaluate the barriers to disseminating the T-EVER to both adolescents and parents. The participants gave a wide variety of responses for the different barriers to sharing the T-EVER with adolescents, but the main concern was that social barriers prevented adolescents from seeking out pharmacist assistance. Specific barriers to the pharmacist’s dissemination of the T-EVER include an adolescent’s previous vaping history, the lack of relationship with the pharmacist, and discomfort when communicating with adults.

Other barriers were identified as well, such as a lack of communication with a pharmacist because adolescents do not visit the pharmacy frequently or do not feel comfortable speaking to the pharmacist because their guardians are present at the time of contact with the pharmacist. These responses largely indicate that adolescents have an interest in learning about vaping, but social barriers prevent them from seeking help from health care professionals like pharmacists.

“If it’s, like, a pharmacist that I don’t really know, then, I’m, like, might be a bit awkward to, like, start a conversation or something.”—Adolescent 1

“Yeah, like, it, it, it might be, like, hard to admit that you did that. Or maybe, like, hard to admit that you want to do that, and that you, like, need, like, help to not try to do that or to stay away from it.”—Adolescent 22

“Teens don’t really go to pharmacies that much. They also might think it’s a little bit awkward talking to a stranger that they don’t know. I feel like it’s a somewhat sensitive topic, like, depending on what they’ve gone through.”—Adolescent 26

#### 3.6.2. Perceived Barriers to Disseminate the T-EVER to Parental Guardians

Adolescent participants typically shared that functional barriers prevented parental guardians from receiving pharmacist intervention to review the T-EVER. The most common of these perceived barriers included a guardian’s lack of time that would be needed to have a productive conversation with the pharmacist. However, some other participants believed that there would be no barriers to guardians receiving pharmacy intervention regarding adolescent vaping education.

On the other hand, a few participants indicated that there may also be some social barriers that would prevent parents from seeking pharmacy intervention. These barriers include guardian vape history, a lack of concern or unwillingness to receive the handout, and the perception that their child does not vape.

“Maybe that if, like, let’s say the parent has a full-time job, and maybe they just don’t like, have enough time to do it.”—Adolescent 12

“Think parents would be very receptive, unless… vaping is something that they struggle with, and then they don’t want to admit that it’s something that needs to be fixed.”—Adolescent 31

### 3.7. Theme 5: Pharmacy Role in E-Cigarette Education

#### 3.7.1. Adolescent Interactions with Pharmacists

Most participants indicated that they have interacted with pharmacists in some capacity. These interactions included both medication pick-up and vaccine administration by the pharmacist. However, most participants noted that when they see the pharmacist, their parents are present for the interaction, indicating a potential barrier to pharmacist intervention. In addition, the participants acknowledged that they infrequently visit the pharmacist, making the possibility of pharmacist intervention a challenge.

“I haven’t had a ton of contact [with the pharmacist], just, because I haven’t needed to, but just picking up prescriptions for if I was sick or whatever. But it’s usually pretty brief.”—Adolescent 24

“I never really talk to pharmacists because my parents usually just ask for the medicine, and then, yeah.”—Adolescent 15

#### 3.7.2. Potential Pharmacist-Led Adolescent Vaping Interventions

Most participants believed that pharmacists could play an integral role in disseminating the T-EVER to adolescents in their community. Many participants indicated that this educational handout could be provided in the form of posters or handouts given at the time of checkout at the pharmacy. The participants also felt that the pharmacist plays an important role in educating youth about vaping and its consequences. They believed that pharmacists could spend time in conversation with adolescents to share information beyond what the T-EVER conveys. Conversely, a few participants indicated that educational sessions led by the pharmacist would be beneficial for this vaping education strategy.

“Having a poster would be a good idea, but then also having the handouts at the front, that they, that people can either take, read right there, or they can even hand it out to people.”—Adolescent 13

“I’d say, like, they can have the flyer there, and then they could also, like, put it in your bag that you get.”—Adolescent 23

“I think that if they could talk in depth about the handout, I think that that would be pretty helpful and would maybe help people to trust information more, I guess.”—Adolescent 24

“Speaking at schools, because I definitely know that schools would be very open to, like, somebody coming and talking to the students about vaping.”—Adolescent 33

## 4. Discussion

The adolescents overwhelmingly shared that an educational handout with embedded resources would be helpful to engage youth in learning about vaping. While most adolescents expressed that they are exposed to vaping, most of them also noted that they do not know as much about the consequences of vaping as they want. Through this study, we noted that adolescents want to be engaged in their health, and most of their peers begin vaping because they incorrectly perceive that the benefits outweigh the harms. Therefore, educational handouts, such as the T-EVER, may help overcome this barrier and better communicate the harms associated with vaping that are typically overshadowed by the positive sentiments shared by friends and family [21].

We also explored how we can maximize adolescent willingness to use this educational handout. Common sentiments that we have heard from the participants to increase youth appeal towards using the T-EVER was to make it attention-grabbing. Adolescents need colors and pictures that draw their attention toward the content of an educational handout. Furthermore, one way we can improve the handout is to add more information that can increase adolescent engagement in their own and their peer’s health. The adolescents shared that they want to learn more about the dangers of vaping, so providing youth with that information could prove beneficial in curbing the trend of the e-cigarette epidemic. If we reach adolescents early before they are drawn to the allure of vaping, there is hope that they can make an informed decision on their own about the dangers of vaping and elicit social change, reversing the normalization of vaping among their peers [27,28].

The pharmacists’ role in addressing adolescent vaping is not yet defined. However, the adolescents in this study shared that there is a need for a trustworthy health care professional to act as a vessel of knowledge to guide youth to better understand the implications of vaping. Nearly all the adolescents were interested in learning more about vaping and its effects on their health, and most of them felt they had received minimal, inadequate, or incorrect information about the harms of vaping. Pharmacists have been shown to play a supportive role in public health outreach and can specifically help support those who vape and prevent others from vaping [19]. The adolescents in this study identified the value of pharmacists along with other health professionals to support their autonomy when learning about the harms and benefits of vaping. Developing an effective intervention for pharmacists to engage adolescents in vaping prevention is of great importance as the rates of youth vaping continue to remain high, and pharmacists can act as a trustworthy source to guide adolescents in their efforts to discern the truth about vaping.

## 5. Limitations

One of the limitations of this study was the convenience sample of the participants, which were all from Wisconsin and predominantly White, which limits the transferability of the findings to more diverse populations of adolescents. Additionally, the participants were all recruited through community pharmacies in Wisconsin, which may have only included adolescents with a closer relationship to their local pharmacy than the average adolescent. However, the intention of this line of research is to examine what potential impact pharmacists could have on adolescent vaping in the communities they reach.

## 6. Conclusions

The pharmacist’s role in adolescent vaping may not be well defined, but there is hope that they can act as a guide to youth, using this educational handout (T-EVER) for combatting misinformation on the health benefits and harms of vaping. The findings from this study will allow for effective revisions to the T-EVER that will further increase youth appeal and receptivity. This handout can be utilized by pharmacists to engage adolescents in conversations that extend beyond the pharmacy and out into the community and the home, supporting the prevention of and reduction in youth vaping. The findings from this study can support a future intervention study to formally evaluate the impact of the T-EVER on adolescent vaping with a larger, more diverse sample of adolescents.

## Figures and Tables

**Table 1:** Participant demographics (n = 35).

Demographic	Frequency	Percent
**Grade in School**		
6th	2	5.7%
7th	10	28.6%
8th	7	20.0%
9th	3	8.6%
10th	4	11.4%
11th	7	20.0%
12th	1	2.9%
Beyond 12th	1	2.9%
**Gender Identity**		
Female	17	48.6%
Male	15	42.9%
Non-Binary	3	8.6%
**Racial and Ethnic Identity**		
White/Caucasian	26	74.3%
Multiple Ethnicities	8	22.9%
Hispanic	1	2.9%

## Data Availability

These data are not publicly available to ensure that participant confidentiality is protected and ethical considerations are upheld.

## Appendix B

**Table A1:** Theme 1: Adolescent knowledge and perceptions of vaping.

Subthemes	Supporting Quotes
**Adolescent Prior Vaping Knowledge**	“I know that it’s, like, it can, like, damage the lungs and, like, mental health of a person… also I learned it can, like, cause lung cancer and lots of health problems.”—Adolescent 1 “I didn’t know much, just that, the e-cigarettes contain nicotine, and nicotine harms the body in—know, it harms the lungs and the brain, but I didn’t really know any specifics.”—Adolescent 28
**Perceived Prevalence of Adolescent Vaping**	“Like, sometimes during the summer, I’ll see kids vaping, and I’ll hear some kids talk about it online like it’s cool. So I think it’s very common.”—Adolescent 3 “Just from talking to people at my school, I, like, talk to a lot of people who do it. I know a lot of people who do it. I’m not necessarily, like, super good friends with them, but, I don’t know, I think it’s pretty common.”—Adolescent 16
**Vaping Culture Among Adolescents**	“They’re either, like, curious about it, or, like, a friend or somebody peer pressures them into doing it.”—Adolescent 30 “I think that it’s harmful to them and that they shouldn’t really do it.”—Adolescent 23

**Table A2:** Theme 2: Adolescent preferences of the T-EVER.

Subthemes	Supporting Quotes
**Desired Platform for the T-EVER**	“I think that people would much rather have it be something where they can just click on a link, and they can go, maybe, to a website and see, like, stuff about it.”—Adolescent 5 “Social media is a big one, because there’s, like, a lot of people on there that will, like, see that more”—Adolescent 14
**Adolescent Use of the T-EVER**	“I think it would be really helpful in the, the way that, like, um, you put the immediate consequences of vaping. Like, not- um, because it’s not just talking about the future, like, it’s the, you know, what could happen right now if you did it.”—Adolescent 16 “Well we talk about it a lot, because we know a lot of people who do, and they like want to make sure, like, that I know, like, the risks and everything and, like, that it’s not okay to do and, like, the punishments that will, like, happen and stuff like that, so. But I feel like it would just kind of be, like, a reminder, just, like, talk about it, even though we already do, but like even more.”—Adolescent 9
**T-EVER Availability in the Community**	“ I think no, no one’s going to go- or I feel like you’d get it across more if you had it in those forced areas, because not as many people are going to, like, go out of their way to research these things.”—Adolescent 5

**Table A3:** Theme 3: T-EVER feedback.

Subthemes	Supporting Quotes
**Positive Feedback**	“And also, I think it’s pretty informative with, like, the percentages of high schoolers that admit to vaping in the past month.”—Adolescent 1
**Negative Feedback**	“I don’t know what it is for everyone, but I feel like it’s pretty easy to get, vapes, so like, I feel like they just need to know, like, how easy it is to get into it, and how, like, once you do the first step, it’s so easy to slip, and just keep continuing, so somehow adding something like that could maybe be useful. Like, how it’s so easy to spiral, kind of downward once you’ve started.”—Adolescent 8

**Table A4:** Theme 4: Barriers to pharmacy-led T-EVER implementation.

Subtheme	Supporting Quotes
**Barriers to Disseminate the T-EVER to Adolescents**	“they’ll be, like, nervous and anxious, because they’re already vaping… it’s going to be hard for them to hear somebody talk about the, the, the bad things that come with vaping, while they are, are vaping”—Adolescent 6 “Might be uncomfortable because your pharmacist is kind of a stranger, and also some people, like, I know, like, don’t like talking in front of their parents about sensitive topics a lot. That might be hard.”—Adolescent 3
**Perceived Barriers to Disseminate the T-EVER to Parental Guardians**	“A bit of a time crunch, and they have to be somewhere that’s really important”—Adolescent 17 “I feel like it can be hard sometimes though, knowing, like- I don’t personally know anyone who vapes, but I know my grandma does smoke, so the topic of smoking is kind of sensitive, because I know she’s also embarrassed about it. But, that topic gets kind of sensitive bringing it up, so I don’t know about some kids at school who might have parents who vape, and saying the risks might be an uncomfortable thing.”—Adolescent 26

**Table A5:** Theme 5: Pharmacy role in e-cigarette education.

Subthemes	Supporting Quotes
**Adolescent Interactions with Pharmacists**	“Whenever I get my flu shots, or any, really shots, that I don’t get it, checkups from a normal doctor, so I don’t know, I probably go in there at least once a year, but I don’t really think it’s much more than twice, three times max.”—Adolescent 8
**Potential Pharmacist-Led Adolescent Vaping Interventions**	“ I think— I think all those are good ideas. I feel like, yeah, when I’m getting a shot, I’m just looking around the room, trying to, like, distract myself from the shot because it hurts worse when you’re, like, “oh, I’m getting a shot.” So if you’re looking at something else, it kind of makes it less painful. So, I feel like I look around and if that’s what I saw, I guess I’d read that probably, you know. And then I think that’s helpful with posters, and then, um, handouts, probably the same thing.”—Adolescent 21 “ Well, they’re pharmacists, they should know, you know, the facts about vaping, and the pros and the cons- which is, there is no pros, by the way. But, I think that it’d be, I think that pharmacists definitely should talk to their- what do you call, like their patients”—Adolescent 6 “During, like, big celebrations throughout town type thing, pharmacies could have like an education type thing, and almost be like, a teaching point, like, some people could volunteer to take the class, get free food”—Adolescent 26
